# A novel method of finding accessory nerve during head and neck surgery

**DOI:** 10.1007/s00405-025-09393-x

**Published:** 2025-05-14

**Authors:** G. Ozyigit, H. B. Cobanoğlu, S. Arslan, A. F. Ozyasar, O. Bahadır, A. U. Isik

**Affiliations:** 1 Department of Otorhinolaryngology, Karadeniz Technical University, Trabzon, Turkey; 2 Department of Anatomy, Karadeniz Technical University, Trabzon, Turkey

**Keywords:** Spinal accessory nerve, Great auricular nerve, Neck dissection, Surgical anatomy

## Abstract

**Introduction and purpose:**

In this prospective study, we performed neck morphological measurements and the relationship between the nerve and important anatomical structures in the neck to prevent spinal accessory nerve (SAN) damage in patients who underwent neck dissection.

**Methods:**

This study was conducted prospectively between October 2021 and September 2023 in 80 patients who underwent neck dissection due to head and neck malignancy.

**Results:**

The mean age of 59.2 ± 17.07 years (18–90 years) of the 80 patients, 54 were men and 26 were women. There was a positive correlation between the distance between the NAM-SAN and the sternocleidomastoid (Scm) muscle thickness(r = 0.242, p = 0.03). There was a positive correlation between the distance between the NAM-SAN and the distance between the mastoid apex and the clavicle midline (r = 0.235, p = 0.036). There was a statistically significant negative correlation between the distance between the NAM-SAN and age (r = − 0.324 p = 0.003). There was a positive correlation between the trapezius-SAN and the distance between the mandibula angulus-clavicle midline (r = -0.243 p = 0.03).

**Conclusion:**

The spinal accessory nerve must be sought for higher than the estimated point In patients which with more scm thickness. It should be considered that as the preoperatively measured mastoid apex-clavicle distance increases, the distance between the NAM and the SAN increases. It should be taken into consideration that the course of the SAN in the posterior triangle will be longer in patients with longer necks. It should be noted that as age increases, the distance between the SAN and NAM decreases.

## Introduction and purpose

The spinal accessory nerve (SAN), the 11th cranial nerve, passes through the inferior aspect of the posterior part of the digastric muscle after entering the neck along with the vagus through the jugular foramen. After entering the scm muscle, the nerve exits the posterior border of the muscle and courses over the levator scapula muscle in the posterior triangle of the neck and reaches the trapezius muscle [1].

Spinal accessory nerve damage may occur in the head and neck region after accidents, obstetric trauma, or radiotherapy. Additionally, iatrogenic or planned nerve damage may occur during neck dissection. The most common cause is damage due to trauma [2].

Head and neck dissections are performed mainly to treat malignancies and metastases in the head and neck region. Due to the long course of the Spinal Accessory Nerve in the neck, the possibility of nerve injury is high during surgery. In spinal accessory nerve damage, shoulder girdle problems such as shoulder pain, a dropped shoulder, and a winged scapula occur clinically. Different types of neck dissection techniques have been developed to prevent symptoms of shoulder dysfunction. Despite all these developments variations in the course of the Spinal Accessory Nerve in the neck continue to be an important problem for clinicians and iatrogenic damage can reach up to 67% in some studies [3].

Although many reference points have been defined to prevent spinal accessory nerve damage during neck dissection, new, reliable, specific, and objective definitions are needed to protect the nerve. In this study, we aimed to define the relationships between the neck morphology of patients and the course of the nerve. We aimed to between the nerve and important anatomical structures in the neck to identify new reference points for easier exploration and protection of the nerve in patients who underwent neck dissection.

## Materials and methods

This study was conducted at the xxx University Ear Nose and Throat Clinic between October 2021 and September 2023. This study was performed in accordance with the principles of the Declaration of Helsinki. Approval was granted by the Ethics Committee of University B (2021/281). Informed consent was obtained from each patient, and the study was explained in detail to every participiant.

Patients older than 18 years who had planned neck dissection due to head and neck malignancy and had a voluntary participation in the study were included. Patients with a previous history of neck dissection, any previous neck surgery, who received radiotherapy to the neck, or who had a history of revision surgery were not included in the study. Patients in whom the accessory nerve was invaded with mass or could not be adequately visualized due to metastasis in the neck were also not included in the study.

**Fig. 1:**
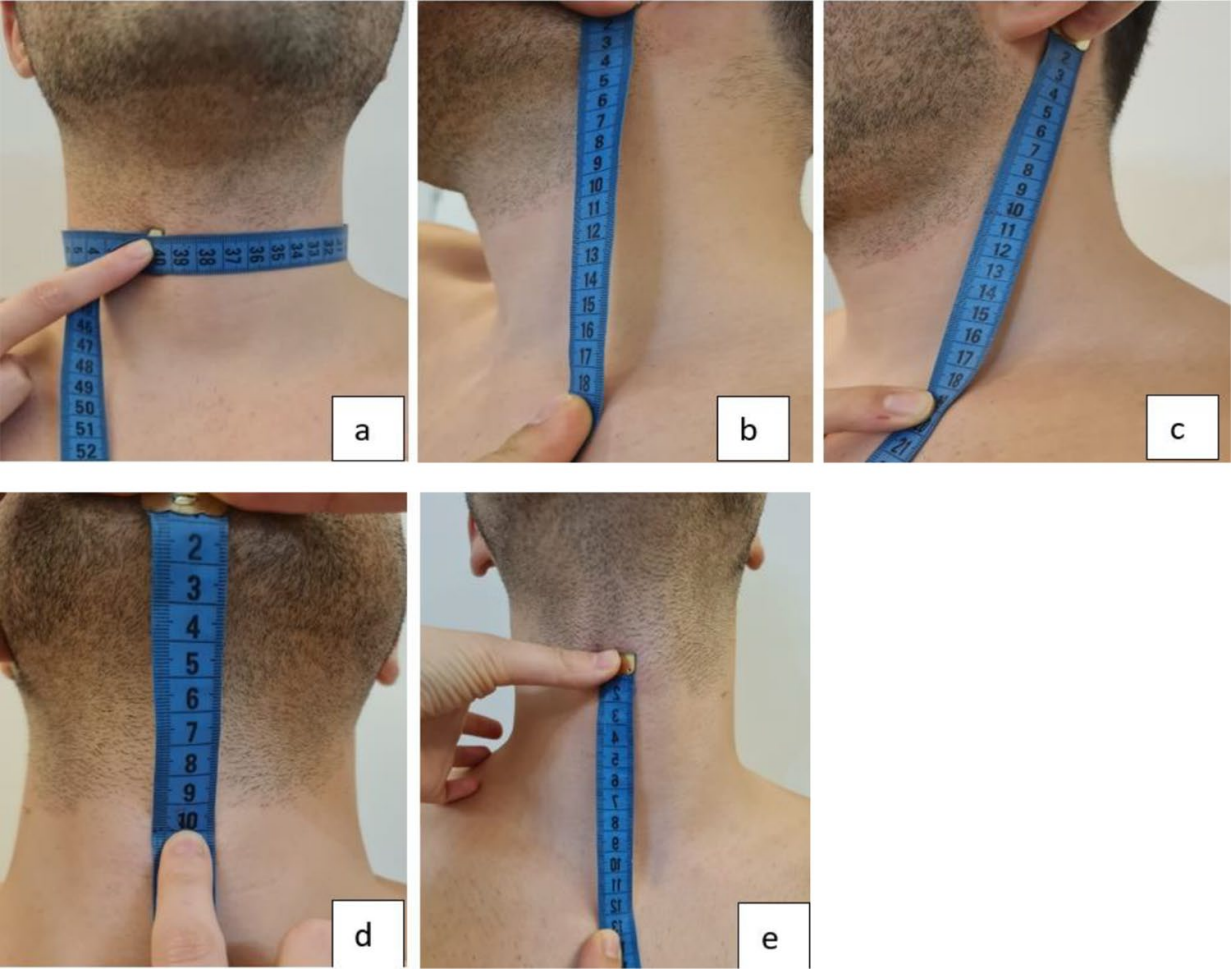
Preoperative measurements; **a** neck circumference from thyroid notch level **b** distance between mandibula angulus—clavicle midline **c** distance between mastoid apex and clavicle midline line **d** distance between the mentum and prominentia laryngea **e** distance between the prominentia laryngea and incisura jugularis

**Fig. 2:**
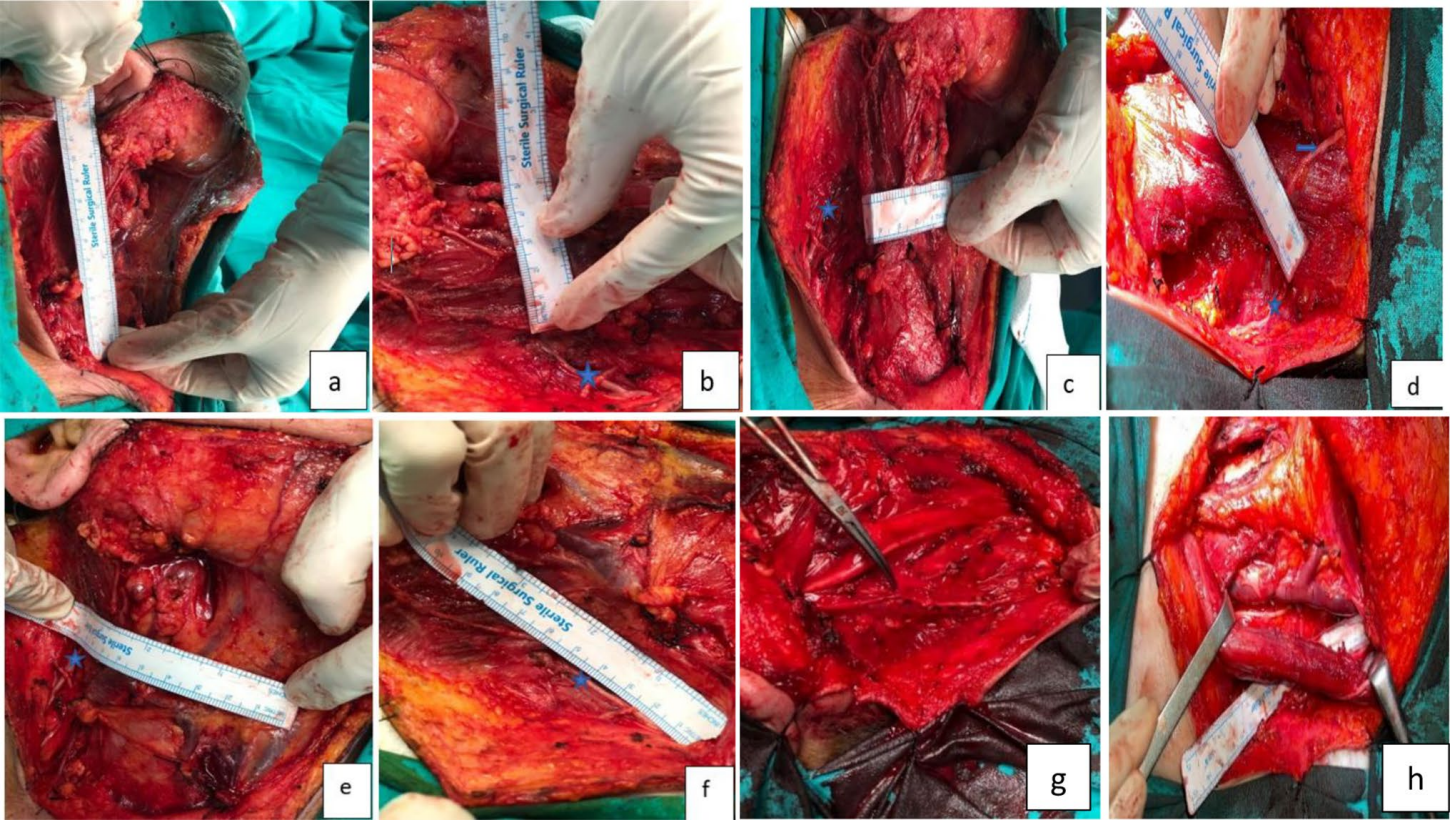
İntraoperative measurements; **a** Distance between mastoid apex origosus and clavicle insertion of Scm muscle, **b** Scm thickness at the point where the SAN exits at the posterior border of the Scm muscle, **c** Scm circumference at the point where the SAN exits at the posterior border of the Scm muscle, **d** At the posterior border of the Scm muscle The distance between the n.auricular magnus (NAM) and the SAN at the point where the SAN exits **e** The distance between the SAN and the thyroid notch at the point where the SAN exits at the posterior border of the Scm muscle, **f** The distance between the SAN and the nerve entering the trapezius muscle at the point where the SAN exits at the posterior border of the Scm muscle, **g**, **h** The distance between the point where the SAN exits the posterior-inferior part of the digastric muscle and enters the trapezius muscle

After providing informed consent, the patients for whom the surgical procedure was planned were seated on the examination table with their neck, back, and shoulders in an anatomical position in the bright examination room. The patient was instructed to look forward and maintain his position during the measurement. Neck topographic measurements and all intraoperative measurements for the side where surgery was planned were made by the same physician for each patient. The same flexible length meter was used for all the measurements. Before the study, forms were created for each patient to be used both in written and electronic form to record preoperative and perioperative measurements. The criteria in Table 1 for preoperative and intraoperative topographic neck measurements were recorded for each patient (Figs. 1 and 2).

**Table 1:** Preoperative and intraoperative measurements

Preoperative measurements	Intraoperative measurements
1. Neck circumference from thyroid cartilage prominentia laryngea(thyroid notch) level	1. Distance between mastoid apex origo and clavicle insertion of scm muscle (SKM length)
2. Distance between angulus mandibula and clavicle midline	2. Scm thickness at the posterior border of the Scm muscle at the point where n.Accessorius exits
3. Distance between mastoid apex and clavicle midline	3. Scm circumference at the point where n.accessorius exits at the posterior border of the Scm muscle
4. Distance between mentum and prominentia laryngea	4. Distance between n.auricular magnus and n.accessorius at the point where n.accessorius exits at the posterior border of the Scm muscle (NAM-SAN)
5. Distance between prominentia laryngea and incisura jugularis	5. The distance between the n.accessorius and the thyroid notch at the point where the n.accessorius exits at the posterior border of the Scm muscle (Thyroid notch-SAN)
	6. The length of N.accessorius at the posterior border of the Scm muscle until it enters the trapezius muscle (Trapez-SAN)
	7. The distance between the posterior belly of the digastric muscle and the part where N.accessorius enters the trapezius muscle (length of the nerve in the neck)

## Statistical analysis

The normality of the distribution of the data was determined by the Kolmogorov‒Smirnov normality test. Descriptive statistics of continuous variables are expressed as the minimum, maximum, and mean ± standard deviation, and distributions of categorical variables are expressed as numbers and percentages. Spearman correlation analysis was performed to evaluate the associations between variables. The relationships were accepted as 0.00–0.19 for no or a negligibly low relationship, 0.20–0.39 for a low level of relationship, 0.40–0.69 for a moderate relationship, 0.70–0.89 for a strong relationship, and 0.90–1.00 for a very strong relationship (38).

P values obtained as a result of biostatistical analyses performed in IBM SPSS 22 and the open source R Studio program environment were evaluated at the α = 0.05 significance level and 95% confidence interval. Comparisons were considered to be statistically significant when p values were less than 0.05.

Experimental (post hoc, retrospective, posterior) power analysis was performed to calculate the realized power in the study. With a significance level of α = 0.05 and an effect size of d = 0.5, the power of the study (1-β) was calculated as 0.99. The power of the sample was calculated in the G*Power 3.0.10 program environment.

## Results

The mean age of 59.2 ± 17.07 years (18–90 years) of the 80 patients, 54 were men and 26 were women. 42 of the 80 necks were on the right side, and 38 were on the left side (Tables 2, 3 and 4).

**Table 2:** Demographic information of the patients

	n	Minimum	Maximum	Mean ± Std
Height (mt)	80	1.49	1.94	1.67 ± 0.09
Weight (kg)	80	41	200	75.91 ± 18.96
Age	80	18	90	59.25 ± 17.07

**Table 3:** Descriptive statistics of patients’ preoperative clinical information

	n	Minimum	Maximum	Mean ± Std
Angulus mandibula-clavicle midline(cm)	80	8	16.5	12.44 ± 1.82
Neck circumference(cm)	80	31	60	38.80 ± 4.61
Mastoid apex-clavicle midline(cm)	80	11	20	15.71 ± 2.05
Mentum-prominentia laringea(cm)	80	4	10	7.5 ± 1.29
Prominentia laringea-incisura jugularis(cm)	80	3.5	10	6.67 ± 1.46

**Table 4:** SCM length: Distance between the mastoid apex and the clavicle insertion site of the SCM

	n	Minimum	Maximum	Mean±Std
SCM length (cm)	80	10	18	14.57±1.51
Trapez-SAN (cm)	80	3	9	5.50±1.39
NAM-SAN (cm)	80	0	3	1.67±0.72
SCM circumference (cm)	80	7	13	10.55±2.05
SCM thickness (cm)	80	3	9	5.35±1.16
Thyroid-SAN (cm)	80	5	15	10.57±1.65
Length on SAN in the neck (cm)	80	6	14.5	10.54±1.85

*Trapez-SAN*: distance between the N. Accessorius at the point where N. Accessorius exits at the posterior border of the SCM and the point where the nerve enters the trapezius muscle. *NAM-SAN*: distance between n.auricular magnus and n.accessorius at the point where n.accessorius exits at the posterior border of the Scm muscle. *SCM circumference*: distance at the point where the N. Accessorius exits at the posterior border of the SCM. *SCM thickness*: distance at the point where the N. Accessorius exits at the posterior border of the SCM. *Thyroid notch-SAN*: distance between the point where the N. Accessorius exits at the posterior border of the SCM and the thyroid notch*. Endurance of the SAN in the neck*: distance between the parts where the N. accessorius enters the trapezius muscle from the medial side of the posterior belly of the digastric muscle.

A positive correlation was found between the NAM-SAN score and Scm thickness (r = 0.242, p = 0.03). There was a positive corelation between the NAM-SAN distance and trapez-SAN distance (r = 0.307, p = 0.006).

No statistically significant difference was found between the distance NAM-SAN distance and neck circumference (r = 0.024, p = 0.835). A positive correlation was found between the NAM-SAN distance and the distance between the mastoid apex and the clavicle midline (r = 0.235, p = 0.036).

A positive correlation was found between the NAM-SAN distance and the length of the spinal accessory nerve, which exits the posterior belly of the digastric muscle and enters the trapezius muscle (r = 0.330 p = 0.003). A statistically significant negative correlation was found between the distance between the NAM-SAN distance and age (r = -0.324 p = 0.003).

A positive correlation was found between the trapez-SAN distance and the distance between the mandibula angulus-clavicle midline (r = − 0.243 p = 0.03). Positive correlations were found between neck circumference and Skm length, Skm circumference, and Skm thickness respectively (r = 0.248, p = 0.026), (r = 0.354, p = 0.001), and (r = 0.346, p = 0.002).

A positive correlation was found between the SCM thickness and the length of the SAN which the depth of the posterior belly of the digastric muscle to the point where the SAN enters the trapezius muscle. (r = 0.345 p = 0.002).

There was a positive correlation between the the all length of the spinal accessory nerve and between the mandibula angulus and the clavicle midline (r = 0.246, p = 0.02). There was a positive correlation between the the all length of the spinal accessory nerve and the distance between the mastoid apex and the clavicle midline (r = 0.327, p = 0.003) (Table 5).

**Table 5:** The results of statistical analysis

	NAM-SAN	Mand-cla	SCM height	SAN lenght	Thyroid notch-SAN
Trapez-SAN					
r	0.307**	− 0.243*	0.453**	0.370**	0.255*
p	**0.006**	**0.03**	**0.001**	**0.001**	**0.022**
N	80	80	80	80	80
Height					
r	0.114	0.206	0.422**	0.154	0.547**
p	0.313	0.067	**0.001**	0.174	**0.001**
N	80	80	80	80	80
Age					
r	− 0.324**	− 0.348**	− 0.188	− 0.134	− 0.470
p	**0.003**	**0.002**	**0.095**	0.235	**0.001**
N	80	80	80	80	80
Mandibula-clavicle					
r	0.154	1	0.272*	0.246*	0.272
p	0.172		**0.015**	**0.028**	**0.015**
N	80	80	80	80	80
Neck circumference					
r	0.024	− 0.057	0.248*	0.001	− 0.146
p	0.835	0.616	**0.026**	0.992	0.197
N	80	80	80	80	80
Mastoid-clavicle					
r	.235*	.654**	.373**	.327**	0.422**
p	**0.036**	**0.001**	**0.001**	**0.003**	**0.001**
N	80	80	80	80	80
SCM lenght					
r	0.317**	0.272*	1	0.317**	0.184
P	**0.004**	**0.015**		**0.004**	0.102
N	80	80	80	80	80
SCM circumference					
r	0.203	0.128	0.238*	0.191	0.004
p	0.07	0.259	**0.034**	0.09	0.974
N	80	80	80	80	80
SCM thickness					
r	0.242*	0.165	0.294**	0.345**	0.027
p	**0.03**	0.145	**0.008**	**0.002**	0.811
N	80	80	80	80	80
Thyroid notch-SAN					
r	0.194	0.479**	0.184	0.025	1
p	0.085	**0.001**	0.102	0.825	
N	80	80	80	80	80
SAN lenght					
r	0.330**	0.246*	0.317**	1	0.025
p	**0.003**	**0.028**	**0.004**		0.825
N	80	80	80	80	80

*Correlation is significant at the 0.05 level (2-tailed)

**Correlation is significant at the 0.01 level (2-tailed)

Bold indicates the p values

## Discussion

The classical method of radical neck dissection, which was first described by Crile in 1906, in which the internal jugular vein, scm muscle and Acc were removed, resulted in Acc damage and poor functional results described as ‘shoulder syndrome’, leading to new searches for nerve preservation. [4]. For this reason, selective neck dissection and modified radical neck dissection have become very popular in recent years. If there is no lymphatic metastasis or if a tumor invades the nerve, there is strong evidence to recommend dissection by preserving the nerve even during radical neck dissection [5–7]. Anatomical landmarks have been defined in the literature for the exploration and protection of the nerve and to minimize iatrogenic injury [8].

The location at which the SAN exits the posterior border of the SCM is an important landmark for protection of the nerve. Many parameters, such as the relationship of this point with the NAM, its relationship with the clavicle and trapezius muscle, its relationship with the thyroid notch, its distance from the mastoid apex, and the ratio of the Scm muscle to its length, have been investigated in the literature [3, 8]. However, there are studies stating that the exit point of this point differs by 75% for the right and left neck [9]. Different results have been reported in different studies regarding these distances. In this study, the relationship between this point and certain structures in the neck was evaluated to explain the variability in the literature and to provide new guiding definitions. The distance of the point where the SAN exits from the posterior of the Scm to the NAM, the relationship of this distance with the thickness of the Scm, the distance to the thyroid notch, and the distance to the point where the SAN enters the trapezius muscle were evaluated.

The distance from the point where the SAN exits the posterior border of the Scm to the clavicle has been investigated in many studies. Kierner et al. reported that the average distance from the point where the SAN exits the posterior border of the Scm to the clavicle is 7–9 cm [9]. Lu et al. reported that this distance was between 5.7 and 12.9 cm in cadaver studies [10]. Tubbs et al. reported that this distance was 6 cm on average and ranged from 4.5–7 cm [11]. This distance was not measured directly in our study. The average Scm length between the intraoperative mastoid apex and the clavicle insertion site of the SCM measured 14.5 ± 1.5 cm, ranging from 10 to 18 cm.

As the Scm length increased, the distance between the NAM and SAN also increased (r = 0.317, p = 0.004). Increasing this distance causes the distance from the SAN to the clavicle to increase. As the distance between the mastoid apex and clavicle midline, measured preoperatively, increased, the distance between the NAM and SAN increased (r = 0.235, p = 0.036). According to the literature, the distance from the point where the spinal accessory nerve (SAN) exits the posterior border of the sternocleidomastoid (SCM) to the clavicle ranges between 5 and 13 cm. In patients with a longer SCM length and a greater preoperative distance between the mastoid process and the clavicle, the SAN should be sought at a higher level. Our study indicates that SCM length and the preoperative mastoid apex-to-clavicle distance are critical measurements for estimating this variation more accurately.”

The Nervus auricularis magnus (NAM) is one of the guiding structures for finding and protecting the SAN during neck dissection. After defining the NAM at the back border of Scm, the location where the SAN at a certain distance from this point exits the back edge of Scm is estimated.

The NAM-SAN distance, one of the most commonly used landmarks, has been investigated in many studies in the literature, and average estimated distances have been reported [12–14]. This distance, which varies between 1 and 3.5 cm on average in the literature, was measured as 1.67 ± 0.72 (0–3) cm in our study. In 78 of the patients, the SAN was superior to the NAM, while in 2 patients, the distance between the NAM and the SAN was 0 cm, meaning that the nerves were explored side by side. While planning our study, we mainly aimed to reveal the reason for the variability in this distance in the literature.

During the planning phase of the study, we hypothesized that as the Scm thickness increases, the distance between the NAM and SAN increases. There are no studies in the literature examining the relationship between the Scm thickness and SAN localization. This is the most unique aspect of our work. In our study, a positive correlation was found between the NAM-SAN and intraoperatively measured Scm thickness (r = 0.242, p = 0.03). As the Scm thickness increases, the distance between the NAM and SAN increases. Our findings suggest that SCM thickness should be considered as a new parameter for accurately estimating SAN localization after skin flap elevation during neck dissection. According to our data, the SAN should be sought at a higher level in patients with greater SCM thickness.”

The mastoid apex-clavicle distance was positively correlated with the intraoperatively measured Scm thickness. The mastoid apex-clavicle distance was also positively correlated with the NAM-SAN distance. As the distance of the mastoid apex from the clavicle, measured preoperatively, increased, both the Scm thickness and the NAM-SAN distance increased. We believe that the preoperative mastoid apex-clavicle distance is an important and new parameter for estimating the location at which the SAN exits from the posterior border of the Scm during neck dissection and for protecting the nerve.

The relationship between the location at which the SAN enters the trapezius muscle and other anatomical structures has been mentioned in many studies in the literature. Kessler et al., using ultrasonography, reported that the estimated length of the SAN in the posterior triangle of the neck in 18 healthy volunteers was 3.5 cm [15]. In studies conducted on cadavers, the length of the SAN in the posterior triangle of the neck was 2–5 cm [16, 17]. In our study, we measured this distance as an average of 5.5 ± 1.38 cm (3–9 cm). Unlike in studies in the literature, we defined this distance as varying according to several variables. This is one of the unique aspects of our work. As the distance between the NAM and SAN increased, the length of the SAN between the point at which it exited the Scm posterior and entered the trapezius muscle increased (r = 0.307, p = 0.006). A positive correlation was found between the preoperatively measured mandibular angle-clavicle midline distance and the length of the SAN in the posterior neck (r = 0.243 p = 0.03). Based on this, it can be predicted that the course of the SAN in the posterior triangle will be longer in patients with longer neck lengths. We believe that as the distance traveled by the SAN in the posterior triangle of the neck increases, the possibility of injury to the nerve in this region increases. Surgeons should be more careful to protect the nerve in patients with long necks. Additionally, these patients should be informed during preoperative planning about the increased risk of nerve injury.

The thyroid notch-SAN distance is one of the most commonly used landmarks for surgeons to estimate where the SAN exits from the posterior edge of the Scm. There are studies indicating that the SAN originates approximately 2 cm above the point where the horizontal line drawn from the thyroid notch intersects the posterior border of the Scm [18]. In our study, the average distance from the point where the SAN originated from the posterior border of the Scm muscle to the thyroid notch was found to be 10.56 ± 1.65 (5–15). This distance is can be affected by many anatomical factors. A positive correlation was found between this distance and the distance between the mastoid apex and the clavicle midline (r = 0.295 p = 0.008). In patients with longer preoperative neck lengths, the point where the SAN exits the posterior border of the SCM should be sought higher than the thyroid notch.

Durazzo et al. reported that the total extracranial length of the SAN varies between 7 and 18.5 cm, and the average length is 12.02 ± 2.32 cm [19]. In our study, we found that the average length of the SAN between the point where it exits from under the digastric muscle and the point where it enters the trapezius muscle was 10.5 ± 1.84 cm (6–14.5 cm). This distance was found to be positively correlated with the Scm length, Scm thickness, and distance between the mastoid and clavicle measured preoperatively (p = 0.004, p = 0.002, and p = 0.003, respectively). As the course of the nerve in the neck becomes longer, the possibility of injury during surgery increases. Therefore, we believe that several preoperative and intraoperative measurements will increase the ease with which surgeons can make predictions about the possibility of nerve injury during nerve dissection. Although performing the preoperative and perioperative measurements we mentioned may seem like a waste of time for experienced surgeons, we believe that it will be useful for junior surgeons. However, in the future, the measurements we mentioned can be measured more practically with computer programs integrated into radiological imaging, and we believe that these measurements can facilitate both navigated and standard surgeries.

## Conclusion

During neck dissection, the possibility of injury to the accessory nerve is quite high due to the long course of this nerve. We think that the reason why the reference points defined in the past were specified in different locations was due to limitations such as the different morphological structures of the patient populations studied, their being in different ethnicities and age groups, and not being performed in homogeneous groups. The most important difference between our study and previous studies on this subject is that our study facilitates the prediction of nerve localization by taking into account the morphological characteristics of the patients It should be kept in mind that the SAN may be more superior in patients with increased SCM thickness and a large preoperative mastoid apex-clavicle distance. It should be taken into consideration that the course of the SAN in the posterior triangle will be longer in patients with longer necks. It should be noted that as age increases, the distance between the SAN and NAM decreases. According to our study in cases where the sternocleidomastoid muscle is thicker, the accessory nerve tends to enter the posterior region of the muscle at a higher level.

## Limitations

Although all measurements were performed by the same physician in our study, this situation can be seen as a limitation of our study considering the variability between observers. This variability can be prevented by using radiological imaging in future studies.

## Data Availability

All data of the patients were created and recorded electronically.
